# Adaptability of shallow subsurface drip irrigation of alfalfa in an arid desert area of Northern Xinjiang

**DOI:** 10.1371/journal.pone.0195965

**Published:** 2018-04-13

**Authors:** Shufang Wang, Xiyun Jiao, Weihua Guo, Jian Lu, Yungang Bai, Liping Wang

**Affiliations:** 1 College of Water Conservancy and Hydropower Engineering, Hohai University, Nanjing, China; 2 Science and Technology Promotion Centre, Ministry of Water Resources, P.R.C, Beijing, China; 3 Xinjiang Institute of Water Resources and Hydropower Research, Ürümqi, China; 4 College of water conservancy, Yunnan Agricultural University, Kunming, China; Estacion Experimental del Zaidin, SPAIN

## Abstract

A suitable irrigation method adopted to arid desert conditions, including a special soil structure and specialized plants, has been continuously studied and improved. A field study was conducted in the Awei irrigation area of Aletai in Xinjiang in 2015 and 2016 to investigate the applicability of shallow subsurface drip irrigation (SSDI) in an arid desert area. A completely randomized block design with three replications and three treatments for drip tape subsurface depths at 5, 10, 20 cm was established. The results indicated that the vertical distribution of the soil moisture of subsurface drip irrigation (SDI, buried depth at 20 cm) was mainly concentrated at 0–60 cm, while SSDI (buried depth at 5 and 10 cm) was concentrated at 0–30 cm. However, the roots distributions were concentrated at 0–30 cm for SDI and SSDI. The chlorophyll content and water consumption intensity for alfalfa first increased and then decreased in arid desert conditions. The dry yield and water use efficiency (WUE) of SSDI (buried depth at 10 cm) were higher than those of SDI. The SSDI was practical in arid desert conditions and the recommended buried depth was 10 cm.

## Introduction

Alfalfa is one of the most important cultivated forage species worldwide. The United States is the world’s largest producer of alfalfa, with an annual value of about one billion dollars [1]. In Mexico, area planted to this specie totals 338,000 ha [2]. In addition, Alfalfa is largely cultivated in Argentina, Canada, Russia, France, and Italy [3]. In Aletai in northwest China, it is one of the five most-common pastorals, and farmers are trying to plant alfalfa in a large arid desert to mitigate the shortage of available forage. Production agriculture in the arid desert predominantly depends on irrigation. It is well known that alfalfa is a large water user [4]. The seasonal crop water requirements for alfalfa is higher compared to those of other drought crops. However, its growth and yield are limited by the shortage of water resources and primitive irrigation methods in arid desert areas.

Subsurface drip irrigation (SDI), which has expanded largely in arid areas in recent years, is a method for reducing the amount of water used to irrigate crops below the soil surface through the use of emitters with discharge rates that are generally in the same range as those of drip irrigation, and SDI appears to have great potential. Generally, the crop yields with SDI were higher than other irrigation methods, such as border irrigation, sprinkler irrigation and surface drip irrigation. Furthermore, SDI requires less water in many cases [5]. The volume of water applied in the SDI treatments used for alfalfa was smaller with respect to amount applied through flood irrigation or sprinklers. However, soil reached its best hydric condition and there was more dry matter [6, 7]. Many previous studies have shown that the fresh and dry yields of alfalfa decreased as the water supply was decreased, whereas the water use efficiency (WUE) increased [8, 9].

A proper SDI, with the optimum lateral depth and lateral space, could enhance crop yields and improve WUEs. Evaluating the effect of lateral distance on crop growth and yield was reported in many previous studies [10, 11]. The appropriate depth is determined by factors such as the soil physical properties, water use, and crop roots. Crop yields were significantly different between different depths. Patel et al. and Santos et al. had reported on the effects of the depth of placement of drip laterals on the yield of potatoes and onions. The depth of placement of drip laterals greatly influenced the shape and dimensions of the wetted area [12–14]. The recommended optimum lateral depth and lateral space are 40 cm and 100–150 cm, respectively, for alfalfa yield [7]. Kandelous et al. provided a framework and evaluated the drip-line depth (20–70 cm) and distance (40–300 cm) [1]. These studies demonstrate that opportunities exist to improve the performance of subsurface drip irrigation systems by adjusting system design to account for differences in depth of placement of drip laterals. However, in previous researches, the lateral depth studied was all almost more than 10 cm [15–17].

Keeping soil moisture content at field-capacity in the root zone tends to maximize crop yield. However, this is challenging in sandy soils with a shallow water table and low water holding capacity [18]. Depth of drip tape depends upon soil type and crop [19]. On some soils of silty clay loam, sandy loam soil and clay sandy loam texture, SDI of alfalfa had been studied mainly at North America [1, 2, 20]. Because of the high gravelly/sandy soil content and strong evaporation of the desert area of Xinjiang, where almost no crops can be planted before, shallow subsurface drip irrigation (SSDI, drip lateral depth is less than 10 cm below the soil surface) is being studied for widely application recently for alfalfa planting. Due to lateral depth of no more than 10 cm, SSDI has advantages of a low installation cost, convenient maintenance and renewal, and there is also no influence of mechanical operation for the drip tapes compared with SDI. Research on the effects of SSDI has been conducted for potatoes and other crops [21]. However, there has almost no study of using SSDI for alfalfa in an arid desert area, especially focus on suitable lateral depth.

The objectives of this study based on field experiments were: (1) To compare SSDI (5, 10 cm) with SDI (20 cm) with respect to the growth, yield and WUE of alfalfa; (2) to determine the reasonable lateral depth of SSDI for alfalfa production and to make a recommendation for alfalfa planting in arid desert conditions.

## Materials and methods

Field experiments were conducted at the Awei experimental station in the county of Qinghe, Aletai city, Xinjiang. The Awei experimental station was a subordinate agency of Water Conservancy Bureau of Qinghe. The study was carried out by Xinjiang Institute of Water Resources and Hydropower Research and Hohai University, it was supported by Science & Technology Department of Xinjiang Uygur Autonomous Region. Therefore, no specific permissions were required for our activities. We confirmed that the field studies did not involve endangered or protected species.

### Experimental location

Field experiments were conducted at the Awei experimental station in the county of Qinghe, Aletai area, Xinjiang. The latitude is 46°25′30″ N and longitude is 90°04'01″E. The climate of the region can be described as a north temperate continental arid climate with a long, cold winter and cool summer. The extreme minimum temperature is -53.0°C, and the maximum temperature is 36.5°C. The annual average temperature is 0°C. The long-term annual evaporation is 1495 mm and precipitation is only 161 mm. The region enjoys only 103 days above freezing. Frosts are common between September and May. The field site soils in this area can be characterized as having a gravelly soil texture with a gravel content that is 22–30% in the 0–30 cm layers, 42–48% in the 30–60 cm layers, and more than 65% below the 60 cm layers (Table 1). The soil composition was determined at the State Key Laboratory of hydrology-water resource and hydraulic engineering. The region has a low organic matter content, and the field capacity is 13.3%. The total effective precipitation, which was equal or greater than 5 mm, was 79 mm and 90 mm during the experimental period in 2015 and 2016, respectively.

**Table 1:** Soil physical properties the experimental zone.

Soil depth, cm	Physical characteristics				Dry bulk density, g.cm^-3^
	Gravel, %	Sand, %	Lime, %	Clay, %	
0~10	22.43	35.98	31.70	9.89	1.65
10~20	28.16	34.44	27.50	9.90	1.78
20~30	27.82	38.28	24.55	9.35	1.77
30~40	42.56	29.49	19.42	8.53	1.82
40~50	48.63	27.61	17.75	6.01	1.84
50~60	48.02	28.81	18.02	5.15	1.88

Note: Gravel>65% at a soil depth below 60 cm.

### Installation of subsurface drip irrigation

The water source for irrigation was pumped from the Qing River in May to September annually, and a seasonal low water level of the river occurred during the remaining months. Internal inlaying drip irrigation tapes made of polyethylene (PE) from Xinjiang Karez Irrigation Technology Limited were used in this experiment. The on-line emitter flow rate was 3.2 L/h, and the spacing was 30 cm. Drip tapes were laid out connected with a main single PE pipe (Diameter 40mm) with separate opening valves for each subplot. The irrigation quota was controlled by a ball valve in each experimental plot.

### Treatments

Three lateral depths were investigated in the current study, including 5 cm below the soil surface and 10 cm below the soil surface in SSDI compared to 20 cm below the soil surface in SDI. The lateral space was 60 cm in all treatments. Drip tapes were buried manually by famers employed. A randomized complete block experimental design with three treatments and three replications was employed. The area for each replication was 72 m^2^ (30 m long and 2.4 m wide).

### Cultural practices

The alfalfa (*Medicago sativa L*. *cv*. *Algonquin)* was sown in 30 cm spaced rows and 15 cm apart from the drip lines using a seeding rate of 52.5kg ha^-1^ on 15 August 2012. The K and P fertilization were injected twice into the irrigation system for a total 20 kg·ha^-1^ ratio of each nutrient per cut, and the N fertilization was 10 kg·ha^-1^.

The irrigation dates for alfalfa in the experiment according to the irrigation regimen are provided in Table 2. The frequency of irrigation was approximately 10 days in the annual irrigation regime schedule, and each cut had 6 irrigation times for a total of 12 times. Each irrigation quota was 37.5 mm.

**Table 2:** Irrigation time of alfalfa.

Year	Cuts	1	2	3	4	5	6
2015	1	May 26	June 5	June 15	June 25	July 5	July 15
2015	2	July 22	August 1	August 11	August 21	August 31	September 10
2016	1	May 15	May 24	June 3	June 13	June 23	July 3
2016	2	July 10	July 20	August 1	August 11	August 21	September 1

Alfalfa was harvested 2 times during the flowering period throughout the year in the experimental field depending on the soil quality, short frost-free period and slow rate of temperature increase; alfalfa was cut to a residual height of 10 cm. Cut 1 occurred on July 20 and July 8, and cut 2 occurred on September 21 and September 10 in 2015 and 2016, respectively. The wet yield was assessed by randomly selected samples, which had an area of 5.4 m2 (6 m long and 0.9 m wide) from each plot. The dry yield was obtained manually by collecting two 0.45 m length and 0.45 m width samples from each plot, which were dried in an oven for approximately 48 h at 75°C or until they reached a constant weight.

### Soil moisture monitoring

The volumetric soil water content of every layer, including the 0–10, 10–20, 20–30, 30–40, 40–60, and 60–100 cm depth ranges, were measured by PR2 (Soil Moisture Profile Probe 2) every 2–5 days in each plot. There were three PR2 probes at three horizontal distances from the drip taps (0, 0.15 and 0.3 m).

Fine roots (<2 mm diameter) were divided into living roots and dead roots by identifying the color and morphology. The color of the living roots is white, and the dead roots are wrinkled, easy to break, and have a darker or brown color. Roots were excavated by soil coring method (10 cm diameter) at flowering stage of every cut. Samples were collected in increments of 10 cm from the surface up to 60 cm depth, while in the horizontal direction from the drip-line of 0, 10, 20, and 30 cm. The soil samples were washed over a sieve under running tap water to remove soil from the roots; then, fine roots were dried at 65°C in an oven until a constant weight was reached.

The chlorophyll content of alfalfa was measured by a SPAD-502PLUS portable chlorophyll meter (KONICAMINOLTA, Japan), which was used on 5 plants that were randomly selected from each plot.

### Crop water use calculation

The soil water use was calculated using the water balance model equation from the soil water profiles measured from:

$$ET_{j}=10\sum_{i=1}^{n}\;H_{i}\left(\theta_{ij}-\theta_{ij+1}\right)+M+P+K-C$$ (1)

where *ET*_j_ is the soil water depletion of stage *j (*mm); *H*_i_ is the thickness of the soil layer (cm); *θ*_ij_ and *θ*_ij+1_ are the soil water content of layer *i* in stages *j* and *j+*1, respectively; *M* is water applied for irrigation (mm); and *P* is precipitation (mm); and the daily precipitation values were obtained from the weather station located at the Awei experimental station. *K* is groundwater recharging (mm), which was considered to be zero, while the located groundwater depth was lower than 3 m. *C* is drainage (mm), which was calculated as *C* = (*PZMC*_j+1_-*PZMC*j) [4], where *PZMC*_j_ and *PZMC*_j+1_ are the soil water content of the percolation zone (60–100 cm) and *C* is set to zero if *PZMC*_j+1_<*PZMC*_j_. When the groundwater table was lower than 3 m below the ground surface, the capillary increase in the groundwater was negligible. The runoff was also ignored because there usually is no runoff in the North China Plain.

The data were statistically analysed with one-way analysis of variance (ANOVA), and treatments means were compared using *LSD* test at *P* = 0.05. All analyses were carried out using IBM SPSS Statistics 22.

## Results and discussion

### Soil water content variety and root distribution in each layer

**Fig 1:**
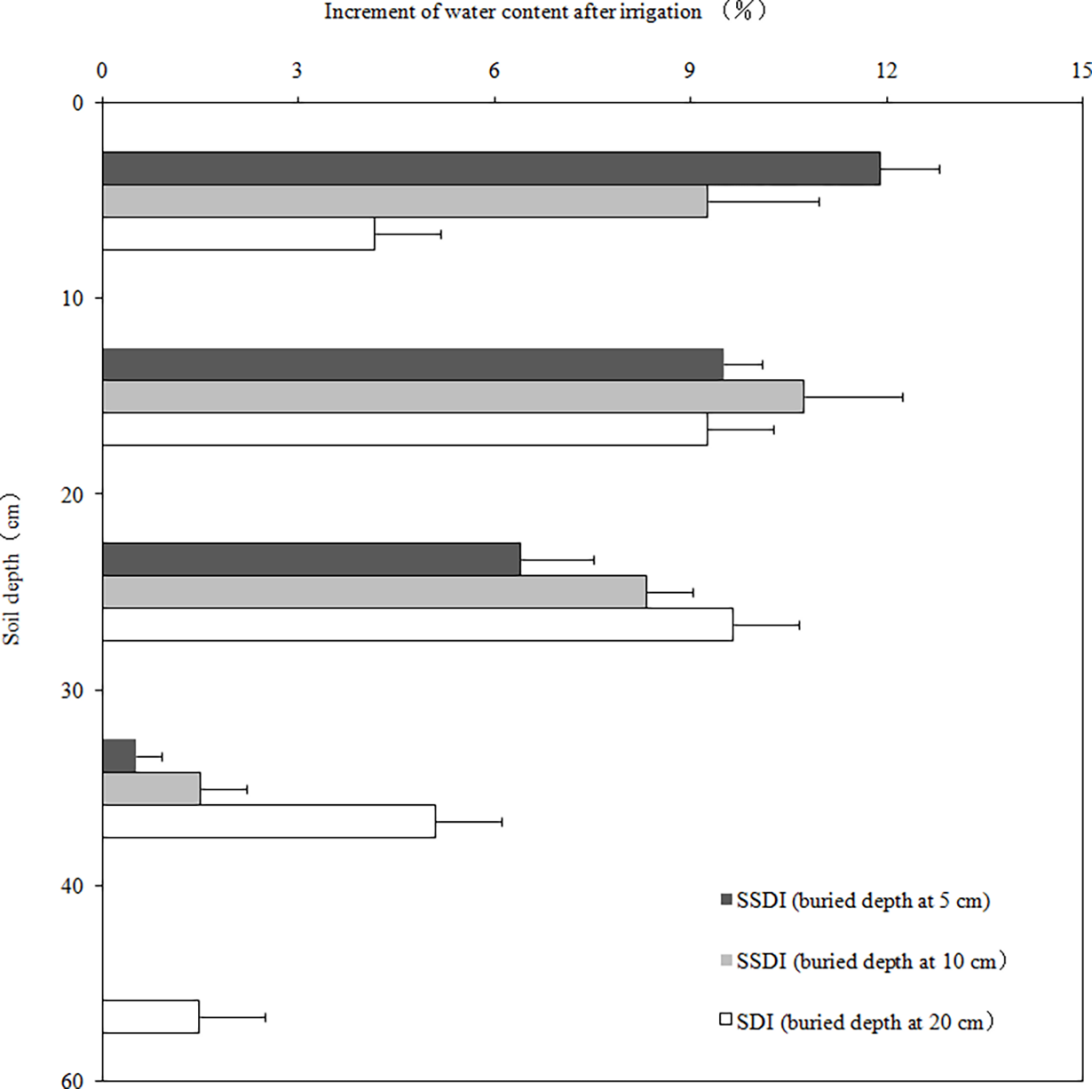
Increment of water content in different soil layers the day after irrigation.

**Fig 2:**
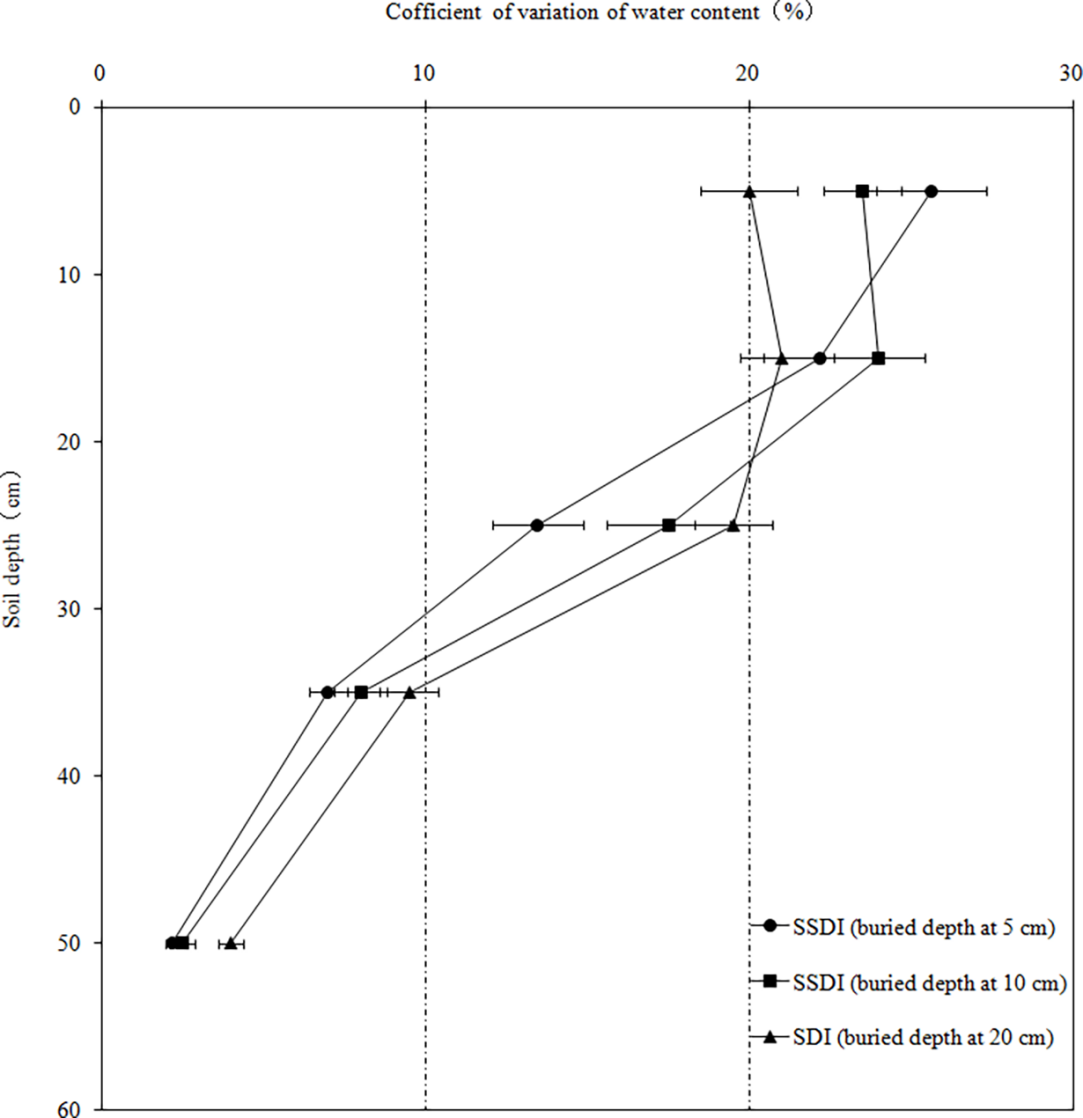
Coefficient of variation of water content in different soil layers (It was stable layer in 60–100cm).

The increment of the soil water content that was highest in the layer of SSDI and SDI was observed in the 0–60 cm layer, especially in the 0–30 cm layer the day after irrigation (Fig 1). The vertical wetting range of SSDI only reached 40 cm underground, while SDI reached 60 cm underground. The variation of soil moisture expressed by the coefficient of variation (*Cv*: standard deviation divided by mean) was divided into 4 levels during the growth period, which were the change layer (*Cv* >30%), active layer (*Cv*: 20%~30%), sub active layer (*Cv*: 10%~20%) and stable layer (*Cv* <10%) [22]. The 0–20 cm layer was active, 20–30 cm layer was sub active, and other layers were stable (Fig 2), primarily because of the influence of the soil texture on the soil water content [23]. The distribution of the soil water content changed due to the special soil properties of the arid desert the day after irrigation, during which the vertical wet depths of SSDI and SDI reached up to 40 cm and 60 cm underground, respectively, which led us to conclude that the soil infiltrability enhanced as the soil layer increased. In addition, the variability of the soil water content in the 0–10 cm layer was highest for all of the treatments, and the surface evaporation was one of the main factors of variability in the soil water content, although gravel soils can reduce evaporation [24].

**Fig 3:**
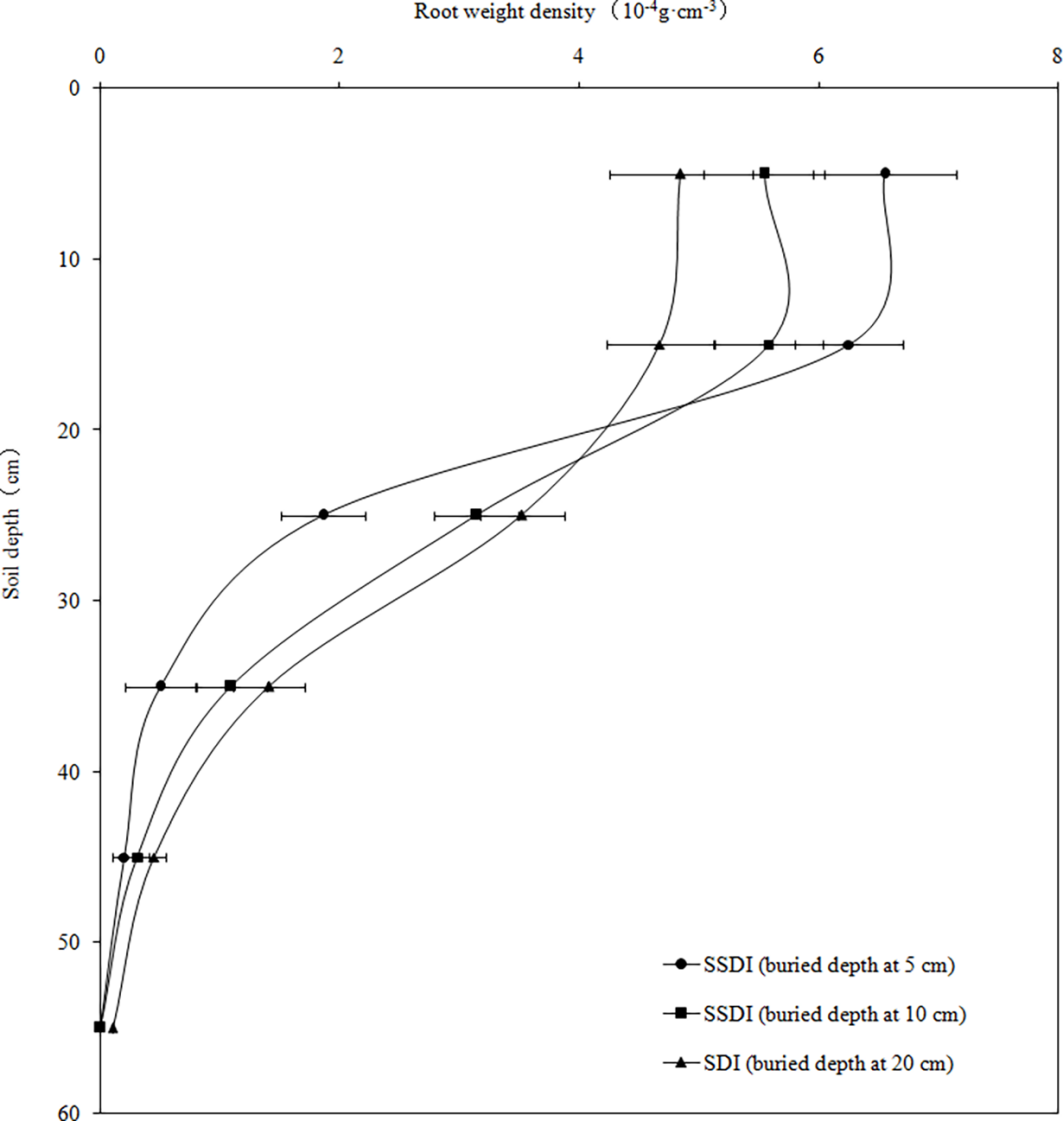
Distribution of root weight density in different soil layers at flowering stage.

**Fig 4:**
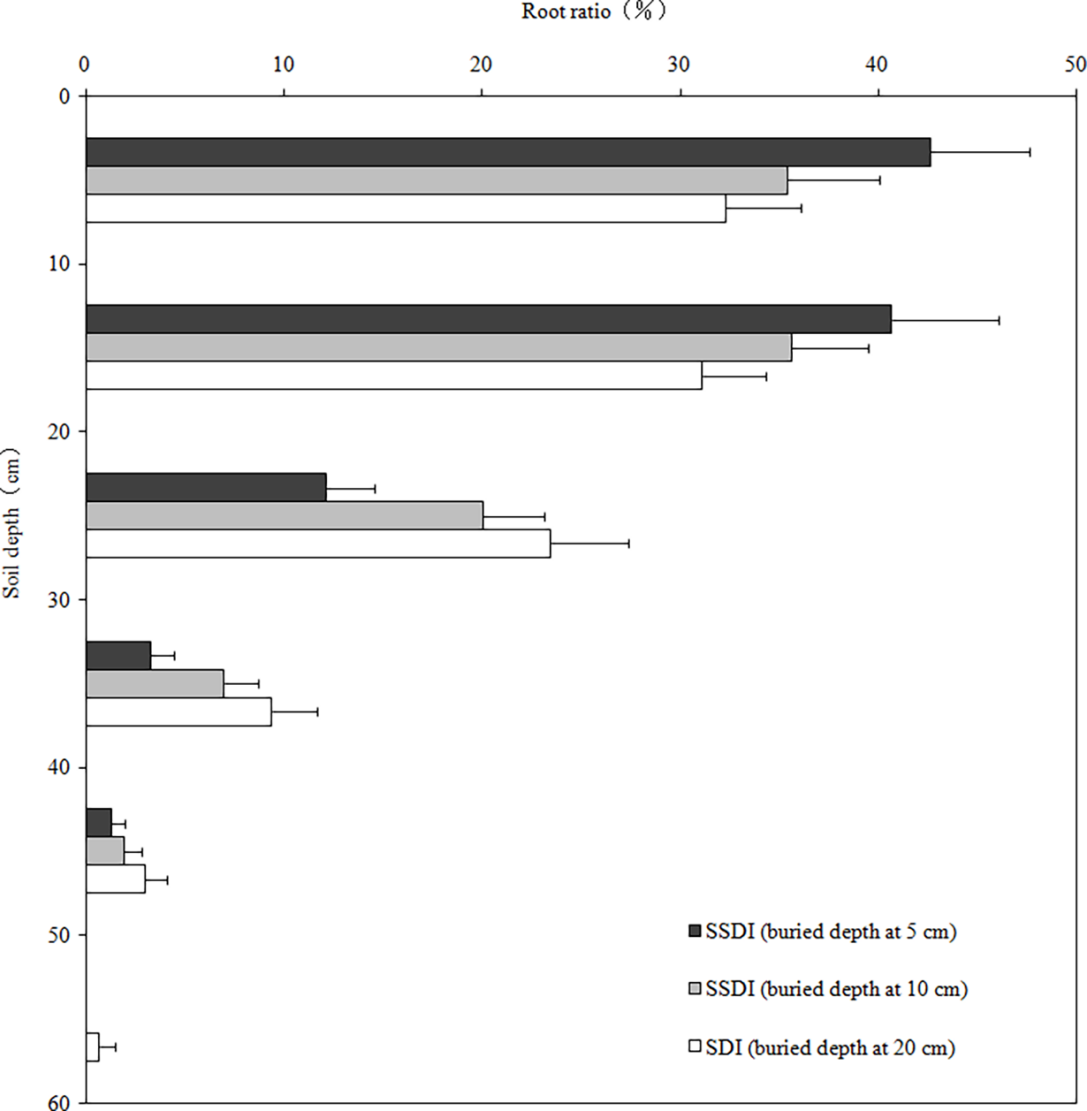
Root weight density ratio in different soil layers at flowering stage of alfalfa.

The factors that affect the biomass of alfalfa roots include the soil layer, soil properties, irrigation, groundwater level and other factors [25]. The distribution of the fine root weight density for the 5 cm and 10 cm depth treatments for SSDI were mainly concentrated in the 0–20 cm and 0–30 cm soil layers, respectively. However, the distribution for SDI was mainly concentrated in the 0–30 cm soil layer. The fine root content was less than 3% for all of the treatments (Figs 3 and 4). This outcome was mainly due to soil obstacles (sticky, compacted and barren). The thinner the depth of the soil layer, the more severe the soil obstacle, which led to a shallower root depth [26]. The rock fragment content of the soil layer within 0–30 cm in the arid desert was much smaller than the soil layer below 30 cm, in which the dry bulk density was greater. Therefore, the root depth of the soil was shallow, and the fine root depth was concentrated in the 0–30 cm soil layer for all of the treatments, which was similar to a study on gravel and extreme phosphorus-deficient plots Fox and Lipps [27]. These results indicated that the higher rock fragment content and bulk density of the arid desert area were not conducive to the growth, penetration and distribution of alfalfa roots, which could affect the water absorption of roots and crop growth.

The influence of the buried depth of the drip tape on the root distribution should be considered in the design of the SDI system [1], and the distribution range of roots for the 5 cm treatments for SSDI was narrower compared to that of other treatments, which showed that the buried depth of drip tape, in addition to the soil texture and structure, had an effect on the root distribution and growth. The root biomass was the largest in the 0–20cm layer, where the drip tape was buried, and this result also indicated that if the root encounters an area with high moisture, it grows and branches because of the hydrotropism of plants [28].

Plants absorb moisture (and other nutrients dissolved in water) from soil, mainly through roots, to meet the needs of their physiological activities, such as growth and metabolism. However, the water absorption process is divided into active water suction and passive water suction. Tall plants absorb water through physical passive water absorption when transpiration is strong, and young plants or tall plants with strongly suppressed transpiration absorb water through physiological active water suction in most cases [29]. Passive water absorption replenishes the loss of moisture caused by transpiration and was the main factor in the growth period due to the strong evaporation in the arid desert area. The greater the soil moisture distribution in the soil layer, the greater the root distribution, leading to an accelerated change in soil moisture [30], which is more conducive to transpiration and the formation of dry matter. Therefore, according to the previous analysis, the change in soil moisture and root distribution of layers in SSDI showed better adaptability to each other.

### Chlorophyll and water consumption intensity in the growth stage

Because of the similar growth of the two cuts, growth and water consumption of alfalfa were studied along with those of second crops. According to the investigation of the plants, the growth stages of the second alfalfa crop were divided into four stages: before the branching stage for approximately 13 days, the branching stage for approximately 23 days, the bud stage for approximately 15 days, and the flowering stage for approximately 11 days.

**Fig 5:**
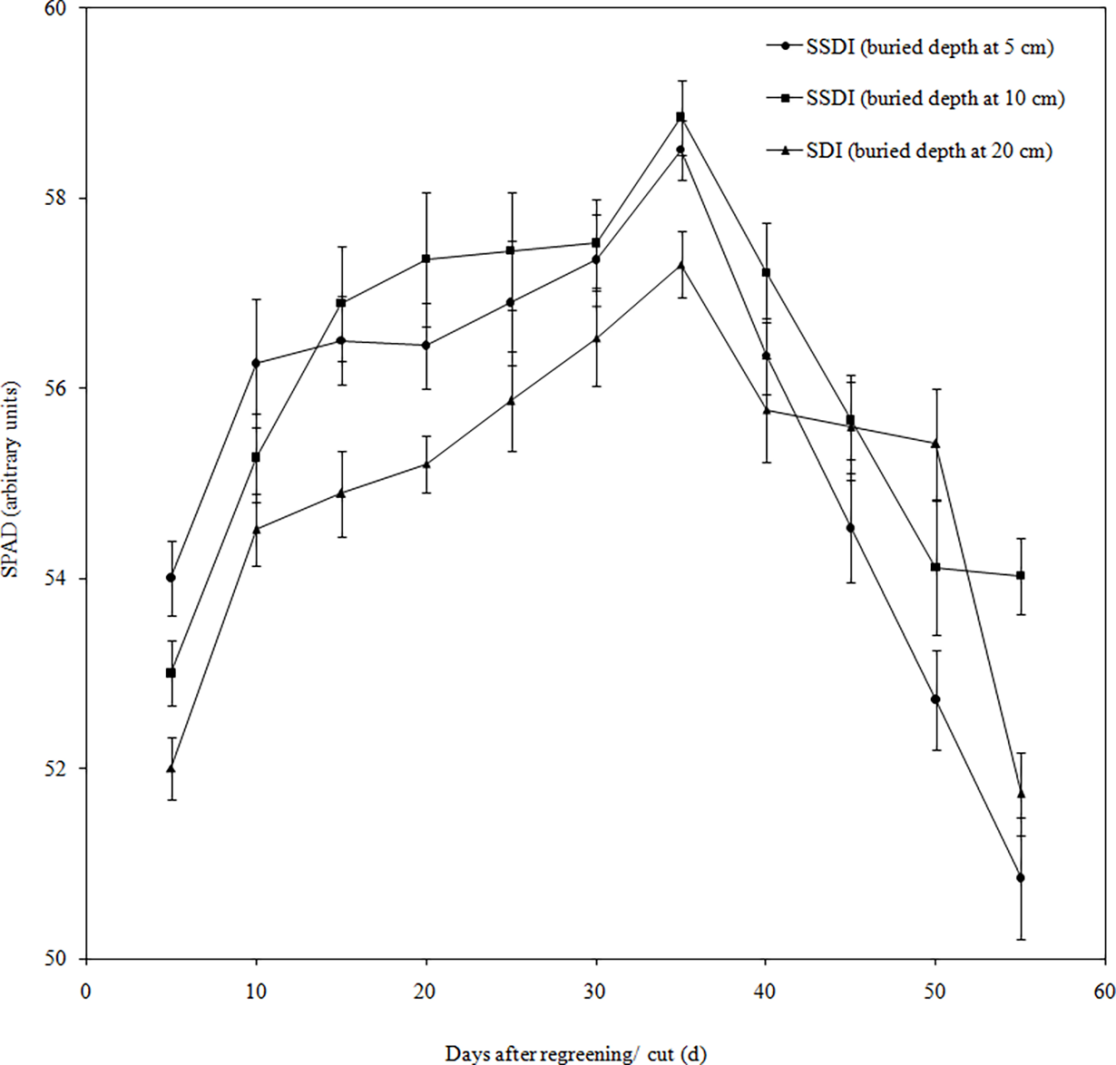
SPAD value of alfalfa during one fertility period.

The relative chlorophyll content can be expressed by the value of soil and plant analyzer development (SPAD) [31]. The trend of SPAD for all of the treatments generally increased steadily until the prime bud stage, during which it achieved its maximum value and then decreased gradually (Fig 5). The SPAD value of the 10 cm treatment of SSDI was slightly higher compared to that of other treatments in general. Chlorophyll is an important pigment that is related to photosynthesis and plays an important role in the absorption, transfer and transformation of light. Chlorophyll can convert light energy into the energy needed for plant growth and development, so the level of chlorophyll also reflects the growth of plants. Previous studies showed that the yield is increased and growth is better when chlorophyll is higher under the same conditions [32]. Therefore, the yield of the 10 cm treatment for SSDI was higher due to the greater amount of chlorophyll in alfalfa.

The trend of water consumption intensity of alfalfa, which was expressed as ET divided by days in stages *j* and *j*+1, generally described as a process that increased at first and then decreased for all treatments during the fertility period. Water consumption was influenced to some extent by the burial depth of the drip tape for every stage (Table 3). Before the branching stage, the water consumption intensity was small, and as a result, transpiration was weak when alfalfa grew slowly during that period. Water consumption mainly came from the surface soil evaporation, the treatments were significantly different between the three treatments. In the branching stage, the water consumption intensity was highest and alfalfa grew vigorously, which required a substantial amount of water for photosynthesis to synthesize dry matter. Water consumption mainly came from the surface soil evaporation and transpiration. The three treatments were significantly different for each other. The water consumption intensity was also high for reproductive growth and nutrition growth at the bud stage and then decreased at the flowering stage when alfalfa grew slowly due to reproductive growth. Water consumption intensity of treatments were also different during the two period stages. The deeper drip type placement depths caused the reduction of evapotranspiration values [33]. Generally, the trend of water consumption of alfalfa during the fertility period in an arid desert region was similar compared to other regions in which water consumption has been studied [34, 35].

**Table 3:** Water consumption intensity under different treatments during one fertility period.

Buried depth (cm)	Water consumption intensity(mm/d)				
	Before branching stage	Branching stage	Bug stage	Flowering stage	Mean value
5	3.43^a^	5.43^a^	4.27^a^	2.46^a^	4.21^a^
10	3.53^a^	5.20^b^	4.34^a^	2.26^b^	4.11^a^
20	3.25^b^	4.87^c^	4.10^b^	2.34^b^	3.89^b^

Note

abc value with the same letter are not different(LSD’s test at *P* = 0.05)

The mean value of water consumption of SSDI was maximal for all the treatments, which was 4.21 mm/d at 5 cm depth. There was no significantly different compared to the treatment at 10 cm depth for SSDI, which was 4.11 mm/d. The mean value of water consumption of SDI treatment was the smallest at 3.89 mm/d. Generally, the water consumption was mainly come from the evapotranspiration (i.e., surface soil evaporation and transpiration). Additionally, the soil moisture was high in the surface layers of 5 cm depth treatment, which lead to more evaporation because of the XinJiang weather. While, as previously was mentioned, the deeper drip type placement depths caused the reduction of evapotranspiration values [33]. Therefore, the treatment of 5 cm lateral depth consumed most water and the treatment of 20 cm lateral depth consumed least water (Table 3). The above information shows that more water was consumed for SSDI compared to SDI. This result was because the moisture in the 0–10 cm soil layer of SSDI was higher, which led to intense evaporation.

### Yield and water use efficiency

Due to almost the same weather, irrigation system, total water applied and field management, Table 4 presents the mean value of dry yield and WUE of the two experimental years under SSDI and SDI. The results indicate that the highest total dry yields were 12740 kg/hm^2^, which was obtained at 10 cm depth for SSDI. The total dry yields were not significantly different from those of the 5 cm depth for SSDI and 20 cm depth for SDI, but they were both significantly lower compared to the 10 cm depth for SSDI. Generally, the depth of the drip tape in the arid desert had an effect on the yield, which was somewhat similar to the report of Kazumba et al., who studied the alfalfa yield at 20 cm and 40 cm depths for SDI at a site that was located a few km southeast of the city of Beer-Sheva, Israel [7]. However, Alam et al. found that the depth of placement had little effect on yield when the site was located south of Garden City, Kansas [15].

**Table 4:** Mean value of dry yield and water use efficiency of the two experimental years.

Buried depth(cm)	Total yield(kg/hm2)			Water use efficient(kg/m3)
	Cut 1	Cut 2	Total	
5	5935^a^	5630^a^	11565^a^	2.24^a^
10	6690^b^	6050^b^	12740^b^	2.51^b^
20	6030^a^	5520^a^	11550^a^	2.40^b^

Note

abc value with the same letter are not different(LSD’s test at *P* = 0.05)

The WUE of alfalfa in an arid desert region for all treatments was in the range of 2.20–2.53 kg/m^3^ (Table 4), which was slightly larger compared to results from other studies, which reported values between 1.13 and 2.35 kg/m^3^ [2, 6, 8, 9]. One of the reasons for this discrepancy might be due to the high gravel content of the soil alleviating evaporation, although evaporation is strong in arid desert regions. Additionally, the temperature gap between day and night was quite large and was favorable to the formation of dry matter even when using the same amount of water. The results indicate that the highest WUE was 2.51 kg/m^3^, which was obtained from the 10 cm depth for SSDI. The lowest WUE occurred at the 5 cm depth for SSDI that was significantly different to the other, which mainly occurred because the soil evaporation was intense when the soil moisture was high in the surface layers.

Because of the absorption and utilization of moisture, growth of root was affected by lateral depth, also the migration and distribution of soil moisture was affected by lateral depth [36, 37], and the dry yield and WUE were different for the treatments. It can be deduced that SSDI was suitable for alfalfa in the arid desert of Xinjiang according to above results in 2015 and 2016. Additionally, compared to many studies in other regions with more than 10 cm lateral depth was recommended, a 10 cm depth for SSDI is recommended in arid deserts, due to the high dry yield and WUE, as well as lower cost of drip taps with shallower buried and easier renewed compared with SDI. The results were depended on the special soil characteristics of arid deserts and special climate conditions in Xinjian. However, the results can provide very useful information to other regions with arid desert.

## Conclusion

Due to the special soil characteristics of arid deserts, the depth of drip taps of SDI had an effect on the vertical distribution of soil moisture and fine roots. The vertical distribution of soil moisture was mainly concentrated in the 0–60 cm layer, while SSDI was concentrated in the 0–30 cm layer the day after irrigation. The root distribution was concentrated in the 0–30 cm layer for all treatments. The variability of soil moisture during the experimental period and root distribution showed some mutual adaptability to each other.

The chlorophyll content of Alfalfa first increased and then decreased in the arid desert. SSDI had a higher chlorophyll content, better growth and greater water consumption intensity than SDI. The dry yield and WUE of alfalfa at the 10 cm depth for SSDI was higher than SDI with same irrigation scheduling, although at 5 cm depth for SSDI was not significantly different from SDI. According to the above research, SSDI was practical in the arid desert.

The results from this study have potentially very important implications for decision makers and alfalfa producers in arid desert regions. In the arid desert, a 10 cm depth for SSDI is recommended. Additionally, the high dry yield and WUE as well as cost of SDI layout labor, maintenance service and renewed labor were higher than those of SSDI, which occurred because of the deeper drip tapes and high gravel content of the soil and made the drip tapes harder to install. The results were depended on the special soil characteristics of arid deserts and special climate conditions in Xinjian. Further work needs to take more treatments considered other factors such as lateral space and irrigation quota in SSDI treatment for alfalfa planting.

## Supporting information

S1 Fig Awei experimental station and experimental plots. (TIF)

S1 Table The growth stages of alfalfa in 2015 and 2016. (PDF)
