# Supplementary figures and images for: Adaptability of shallow subsurface drip irrigation of alfalfa in an arid desert area of Northern Xinjiang
Source: PLoS One. 2018 Apr 13;13(4):e0195965. doi: 10.1371/journal.pone.0195965 (PMC5898749; doi:10.1371/journal.pone.0195965)

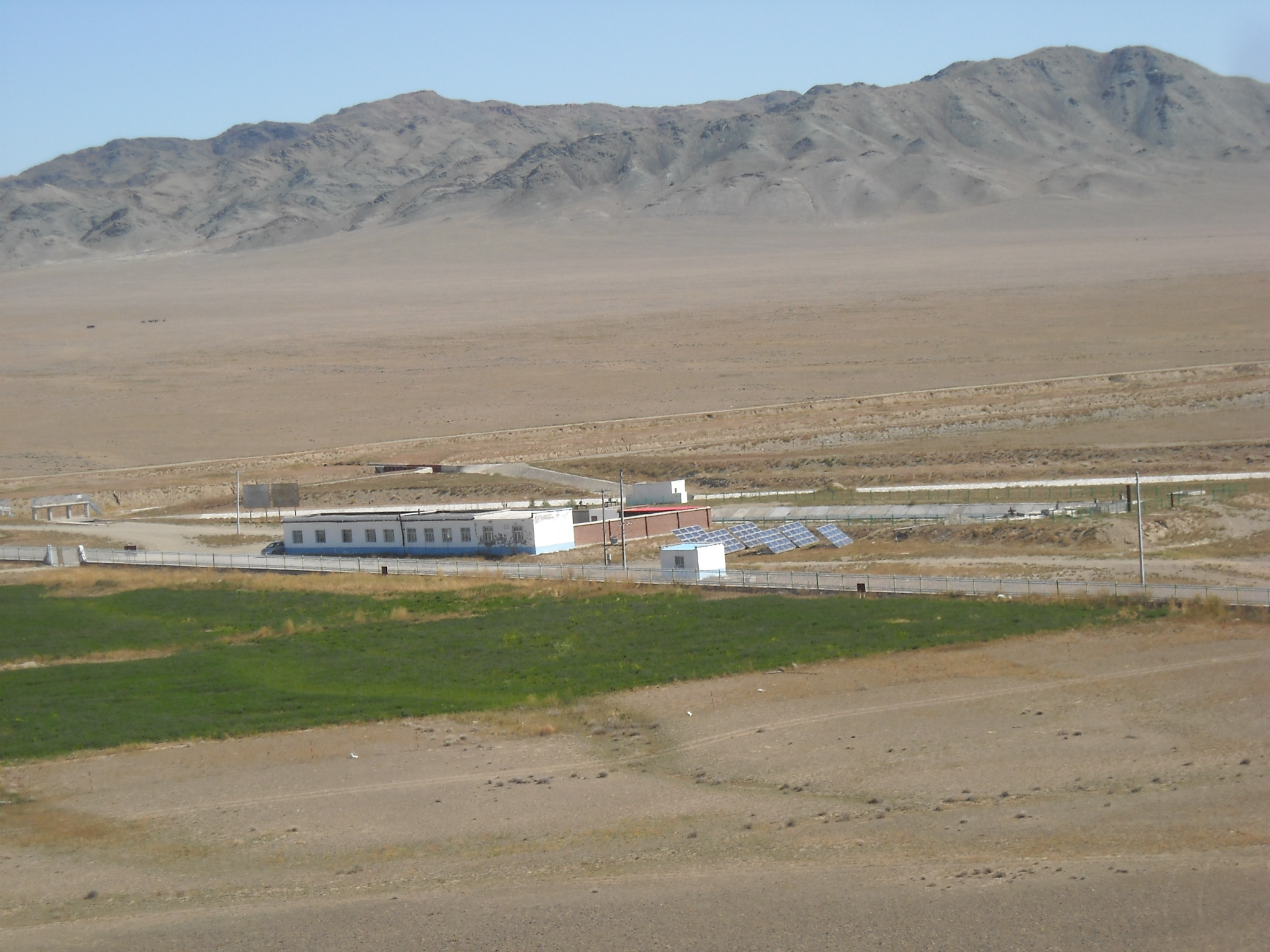

Supplement: S1 Fig — (TIF) [file pone.0195965.s001.tif]
