# Supplementary material for: Adaptability of shallow subsurface drip irrigation of alfalfa in an arid desert area of Northern Xinjiang
Source: PLoS One. 2018 Apr 13;13(4):e0195965. doi: 10.1371/journal.pone.0195965 (PMC5898749; doi:10.1371/journal.pone.0195965)
Supplement: S1 Table — (PDF) [file pone.0195965.s002.pdf]

**S1 Table. The growth stages of alfalfa in 2015 and 2016**

| Year | Cuts | Before branching stage | | Branching stage | | Bug stage | | Flowering stage | |
|---|---|---|---|---|---|---|---|---|---|
| 2015 | 1 | 19-May | 3-Jun | 4-Jun | 26-Jun | 27-Jun | 8-Ju1 | 9-Ju1 | 20-Ju1 |
| | 2 | 21-Ju1 | 1-Aug | 2-Aug | 23-Aug | 24-Aug | 8-Sep | 9-Sep | 21-Sep |
| 2016 | 1 | 8-May | 20-May | 21-May | 14-Jun | 15-Jun | 27-Jun | 28-Jun | 10-Ju1 |
| | 2 | 11-Ju1 | 22-Ju1 | 23-Ju1 | 14-Aug | 15-Aug | 28-Aug | 29-Aug | 10-Sep |
